# Cognitive Decline in Alzheimer’s Disease: Limited Clinical Utility for GWAS or Polygenic Risk Scores in a Clinical Trial Setting

**DOI:** 10.3390/genes11050501

**Published:** 2020-05-02

**Authors:** Jack Euesden, Sivakumar Gowrisankar, Angela Xiaoyan Qu, Pamela St. Jean, Arlene R. Hughes, David J. Pulford

**Affiliations:** 1 GlaxoSmithKline Medicines R&D, Stevenage, Hertfordshire SG1 2NY, UK; david.x.pulford@gsk.com; 2 Parexel International, 2520 Meridian Parkway, Durham, NC 27713, USA; Sivakumar.Gowrisankar@parexel.com (S.G.); Angela.Qu@parexel.com (A.X.Q.); Pam.StJean@parexel.com (P.S.J.); Arlene.Hughes@parexel.com (A.R.H.)

**Keywords:** Alzheimer’s disease, Alzheimer’s disease late onset, polymorphism, single nucleotide, genetic variation, dementia, apolipoprotein E4, risk factors, genome-wide association study

## Abstract

*Introduction*: Alzheimer’s disease (AD) is a progressive and irreversible neurological disease. The genetics and molecular mechanisms underpinning differential cognitive decline in AD are not well understood; the genetics of AD risk have been studied far more assiduously. *Materials and Methods*: Two phase III clinical trials measuring cognitive decline over 48 weeks using Alzheimer’s Disease Assessment Scale-cognitive subscale (ADAS-cog, *n* = 2060) and Clinical Dementia Rating-Sum of Boxes (CDR-SB, *n* = 1996) were retrospectively genotyped. A Genome-Wide Association Study (GWAS) was performed to identify and replicate genetic variants associated with cognitive decline. The relationship between polygenic risk score (PRS) and cognitive decline was tested to investigate the predictive power of aggregating many variants of individually small effect. *Results*: No loci met candidate gene or genome-wide significance. PRS explained a very small percentage of variance in rates of cognitive decline (ADAS-cog: 0.54%). *Conclusions*: These results suggest that incorporating genetic information in the prediction of cognitive decline in AD currently appears to have limited utility in clinical trials, consistent with small effect sizes estimated elsewhere. If AD progression is more heritable soon after disease onset, genetics may have more clinical utility.

## 1. Introduction

For the past 10–20 years, Alzheimer’s Disease (AD) drug development has unsuccessfully focused on disruption of β-amyloid and tau pathology, intervening by halting or reversing visible AD damage in symptomatic patients. More recently, the AD field has shifted its focus toward molecular targets (e.g., TREM2 [1] and CD33 [2]) aimed at preventing β-amyloid and tau pathologies in presymptomatic patients before cognitive impairment occurs or in patients with mild cognitive impairment before irreversible neuronal damage occurs. 

The major genetic risk factor for late-onset AD (LOAD) is *APOE* with carriers of the ε4 allele at increased risk for AD [3]. While the most recent genome-wide association study (GWAS) of AD identified 29 susceptibility loci for risk [4], few examples exist of loci associated with cognitive decline in AD once the disease has manifested itself [5,6]. To address this gap, data from two completed clinical trials were used to evaluate genetic association with AD disease progression using candidate variants, GWAS, and polygenic risk score (PRS) approaches.

## 2. Materials and Methods 

### 2.1. Study Population

The analysis population was derived from two 54-week placebo-controlled parallel-group phase III studies AVA102670 and AVA102672 [7] designed to investigate the effect of rosiglitazone maleate extended release (XR) on cognitive decline. The genetic analysis population was drawn from the Intent to Treat (ITT) population: all randomised subjects who received at least one dose of investigational product/placebo and met the inclusion/exclusion criteria. The clinical trials failed to show a benefit of rosiglitazone over the placebo; therefore, subjects were pooled across the treatment arms for this analysis. The analysis was restricted to patients diagnosed with mild to moderate AD who provided written informed consent for genetic research, a DNA sample, and were successfully genotyped. Analysis was also restricted to White/Caucasian/European patients to mitigate confounding due to population-specific differences in genotype frequency. Ethics approval for this genetic analysis and oversight were provided by the Advarra Institutional Review Board (sponsor protocol approval notice: Pro00028405) on 10 July 2018.

### 2.2. Phenotype Data

Change from baseline at week 48 in two instruments were evaluated as cognitive decline phenotypes: Alzheimer’s Disease Assessment Scale-cognitive subscale (ADAS-cog) and Clinical Dementia Rating Scale-Sum of Boxes (CDR-SB) scores. In the ITT population, treatment began at week 0 and completed at week 48. ADAS-cog testing was performed at weeks 0, 8, 16, 24, 36, and 48. CDR-SB testing was performed at weeks 0, 12, 24, 36, and 48 (Figure S1). Consistent with the clinical study analyses, the last observation carried forward (LOCF) values were used for missing week 48 subject data for both endpoints. 

### 2.3. Regression of Genotypes by Phenotypic Data

Full details of genotyping, quality control, and specific candidate variants investigated are summarised elsewhere (Table S2, Figure S2, and Data S7). Residualised phenotypes were generated in R version 3.5.1 by regressing change in each endpoint on the first six genetic principal components (PCs), study, *APOE* ε4 copy number, and clinical covariates: screening MMSE (Mini Mental State Examination) score, baseline BMI, treatment, years of education, time on treatment, and corresponding test baseline scores (i.e., ADAS-cog or CDR-SB). These residualised phenotypes were regressed on genome-wide genotypes and 39 candidate Single Nucleotide Polymorphisms (SNPs) (Table S2 and Figure S2) using linear regression. The candidate SNPs were derived from literature and previous GWAS (Table S2a,b).

### 2.4. Construction of Polygenic Risk Scores 

PRSs were calculated for all subjects using the results of an existing GWAS for AD risk in a case-control setting [8]. For power calculations see S5. Scores were calculated using genome-wide data, including the region around *APOE,* at 10,000 *p*-value thresholds from 0.0005 to 0.5 in increments of 0.00005 plus an additional threshold at 5 × 10^−8^ to investigate the predictive ability of GWAS hits alone [9]. At each threshold, each endpoint was regressed on each subject’s PRS, the clinical covariates used for primary genetic association analysis, and six PCs. A significance threshold of 0.001 was used for investigating PRS across multiple thresholds, as proposed previously [9].

### 2.5. Genome-Wide Complex Trait Analysis

Genome-wide Complex Trait Analysis (GCTA) [10] was used to estimate the SNP heritability of each cognitive decline endpoint. This method uses a mixed model approach to estimate the proportion of variance in phenotype that can be explained by genetic similarity between individuals. It can be thought of as providing an upper limit of the variance that can be explained by PRS. A mixed model was fit in GCTA using expectation maximization with a maximum of 10,000 iterations, with fixed effects of the clinical covariates also used in the PRS analysis and six PCs.

## 3. Results

### 3.1. Subjects 

Subjects in the genetic analysis population are representative of the White ITT population with respect to demography and efficacy endpoints (Table 1). 

### 3.2. Association Analysis Results 

Phenotype is residualised and transformed prior to association analysis. The effect of each variable used in residualisation is summarized in Table S4. Treatment duration and MMSE were significantly associated with both endpoints. Years of education and BMI were associated with ADAS-cog change but not CDR-SB change; baseline CDR-SB is associated with CDR-SB change, but baseline ADAS-cog is not associated with ADAS-cog change. For both ADAS-cog and CDR-SB endpoints, there was a nominal but nonsignificant (α = 6.41 × 10^−4^) association between *APOE* genotype (number of ε4 alleles) and cognitive decline (*p* = 0.019 and *p* = 0.033, respectively).

**Figure 1:**
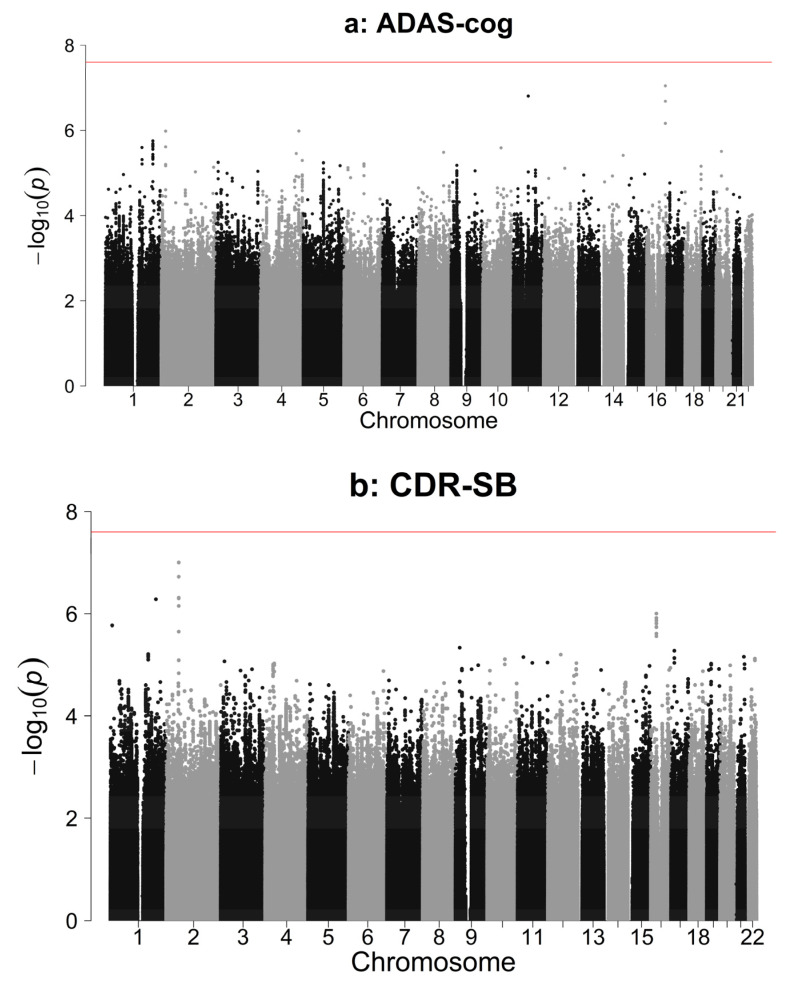
Manhattan plots showing Genome-Wide Association Study (GWAS) results for two cognitive decline endpoints. Red line: GWAS significance threshold of *p* < 2.5 × 10^−8.^

There were no significant associations with any of the 39 candidate variants or genome-wide variants with either primary endpoint (Figure 1, Table S2a,b and Data S3). Significance thresholds are Bonferroni corrected. α = 0.05 was split equally between candidate variants and genome-wide variants analyses. The resulting α = 0.025 was further split between 39 candidate variants, equaling a threshold of α = 6.41 × 10^−4^. For the genome-wide analysis, the conventional *p*-value threshold of 5 × 10^−8^ was halved, resulting in α = 2.5 × 10^−8^.

### 3.3. Polygenic Risk Scores

For the two endpoints ADAS-cog and CDR-SB, the most predictive *p*-value threshold (P_T_) for genome-wide PRSs was identified based on variance explained; these thresholds (*P_T_* = 0.0707 and *P_T_* = 0.00005 respectively) are determined by the data to give the most predictive score. PRS explains a small, statistically significant (*p* = 0.0004) proportion of variance (0.54%) in ADAS-cog change (Table 2). Alzheimer’s PRS did not significantly predict CDR-SB change (*p* = 0.08) (Table 2 and Data S9). A 1-standard-deviation increase in Alzheimer’s PRS would correspond to a 0.53 point change in ADAS-cog and a 0.09 point change in CDR-SB. An interaction analysis for ADAS-cog for 39 AD risk variants and PRS showed no significant interaction (Data S10).

### 3.4. GCTA Results

The heritability estimate for ADAS-cog change was 2.47% (SE = 8.83%). The heritability estimate for CDR-SB change was 8.26% (SE = 10.24%). These were very low heritabilities estimated with wide confidence intervals and small point estimates; for example, Yang et al [10] reported heritabilities for Crohn’s Disease, bipolar disorder, and type 1 diabetes as 56% (SE = 7%), 71% (SE = 7%), and 57% (SE = 7%), respectively. These results from GCTA suggest that the heritability of cognitive decline is low—albeit the power for GCTA, even to detect large heritabilities, was poor in this sample (power < 30% for heritability of 20%, Table S6). 

## 4. Discussion

No genetic variants identified met the threshold for statistical significance for either endpoint. This result is consistent with previous findings that common and rare genetic variants for AD susceptibility have a limited impact on the rate of cognitive decline in AD patients [6] (Table S8). It must be noted that the sample size for disease progression studies, including the present one, are much smaller than those in AD risk GWAS.

The present analysis investigated cognitive decline in AD patients with mild-to-moderate disease and showed limited evidence for a genetic component in this phenotype. This finding may not generalize to cognitive decline in AD generally, and instead, the lack of genome-wide significant loci, poor predictive power of PRS, and low heritability as measured by GCTA may be due to characteristics of the current study population. These include the disease stage; relatively short, 48-week, follow-up period; and the strictly defined clinical trial population, all of which may mask the influence of any genetic factors on cognitive decline. Alongside the relatively small sample size and homogeneous patient characteristics, the latter being consistent with interventional drug trials, these aspects of the study population represent the main limitations.

The poor predictive ability of PRS in AD cognitive decline seen here is consistent with results from GCTA and GWAS. There was no evidence for large effect loci in GWAS or for either progression phenotype having a high heritability as measured by GCTA. Low heritability and variants of individually small effect suggest that, even with larger, better powered Alzheimer’s GWAS or enhanced risk scoring methods, genetics is unlikely to provide substantial utility in differentiating fast progressors from slow progressors in patients with mild to moderate but advanced disease. 

## Figures and Tables

**Table 1:** Demographic data across treatment arms in Intent to Treat (ITT) and genotyped populations: Percentages may not total 100% due to rounding.

Measure	ITT	Genotyped
Randomised treatment, Pooled Group, *n* (%)	2532 (100%)	2164 (100%)
Sex, F:M (%F)	1447:1085 (57%)	1234:930 (57%)
Age, Years, Mean (SD)	73.71 (8.14)	73.66 (8.09)
Ethnicity *n* (%)		
Hispanic or Latino	284 (11%)	249 (12%)
Not Hispanic or Latino	2238 (88%)	1910 (88%)
Missing, *n* (%)	10 (<1%)	5 (<1%)
ADAS-cog change from baseline W48 LOCF, Mean (SD)	2.98 (6.71)	2.94 (6.78)
Missing, *n* (%)	99 (3.9%)	71 (3.3%)
CDR-SB change from baseline W48 LOCF, Mean (SD)	1.50 (2.46)	1.47 (2.45)
Missing, *n* (%)	196 (7.7%)	149 (6.9%)
Baseline ADAS-cog, Mean (SD)	24.65 (9.86)	24.49 (9.86)
Missing, *n* (%)	5 (<1%)	4 (<1%)
Baseline CDR-SB, Mean (SD)	6.79 (3.52)	6.73 (3.52)
Missing, *n* (%)	41 (1.6%)	32 (1.5%)
BMI, Mean (SD)	25.65 (4.03)	25.68 (4.01)
Missing, *n* (%)	6 (<1%)	4 (<1%)
Number of copies of *ApoE*4, Mean (SD)	0.71 (0.69)	0.72 (0.69)
Screening Mini Mental State Examination (MMSE), Mean (SD)	19.78 (4.11)	19.85 (4.08)
Missing, *n* (%)	2 (<1%)	2 (<1%)
Years of Education, Mean (SD)	10.76 (4.02)	10.76 (4.03)
Missing, *n* (%)	7 (<1%)	4 (<1%)
Time in treatment (days), Mean (SD)	296.86 (88.23)	299.93 (84.73)
Missing, *n* (%)	26 (1%)	21 (<1%)

ADAS-cog: Alzheimer’s Disease Assessment Scale-cognitive subscale, CDR-SB: Clinical Dementia Rating Scale-Sum of Boxes, LOCF: Last Observation Carried Forward.

**Table 2:** Predictive ability of PRS on cognitive decline: The *p*-value threshold, P_T_, is the optimum *p*-value threshold for selecting SNPs and weights from Lambert et al. [8] to produce the most predictive PRS.

Phenotype	ADAS-cog	CDR-SB
Threshold P_T_	0.0707	0.00005
Variance explained by PRS (*R*^2^)	0.0054	0.0014
Variance explained by all covariates (*R*^2^)	0.1172	0.1034
Variance explained by all covariates other than PRS—“Null Model” (*R*^2^)	0.112	0.1021
Effect of 1 SD increase in PRS on cognitive decline	0.529	0.092
Coefficient for PRS	3646.94	11.99
Standard Error for PRS	1026.53	11.99
*p*-value for |Coefficient| = 0	0.0004	0.0817
Number of SNPs in PRS (<P_T_)	15960	93

## Data Availability

The GSK sponsored summary statistics generated and/or analysed during the current study can be requested by making an enquiry via www.clinicalstudydatarequest.com.

## Supplementary Materials

The following are available online at https://www.mdpi.com/2073-4425/11/5/501/s1, Supplementary 1: Trial Design; Figure S1. Study Schematic; Supplementary 2: GWAS Results; Table S2a. Candidate Variant Analysis: Change from Baseline in ADAS-cog at Week 48; Table S2b. Candidate Variant Analysis: Change from Baseline in CDR-SB at Week 48; Figure S2a. QQ plot for ADAS-cog; Figure S2b. QQ plot for CDR-SB; Supplementary 3: Power Calculations for Association Studies; Figure S3. Power curves for candidate and GWAS analysis, for both endpoints; Supplementary 4: Detail on Clinical Covariates; Table S4a. Effect of Covariates in GWAS: ADAS-cog; Table S4b. Effect of Covariates in GWAS: CDRSB; Supplementary 5: Power Calculations for PRS; Figure S5a. Power Curves for PRS predicting ADAS-cog change; Figure S5b. Power Curves for PRS predicting CDR-SB change; Supplementary 6: Power Calculations for GCTA; Table S6. Power to detect different heritability values; Supplementary 7: Details on Genotyping and QC; Supplementary 8: Mixed Model Analysis; Table S8. Results of individual candidate SNP association with progression as assessed by a mixed model with repeated measures; Supplementary 9: Quantiles Plots; Figure S9. Quantiles plots; Table S9. PRS Quantiles; Supplementary 10: Interaction Analysis between PRS and Candidate Variants.
