# Supplementary material for: Cognitive Decline in Alzheimer’s Disease: Limited Clinical Utility for GWAS or Polygenic Risk Scores in a Clinical Trial Setting
Source: Genes (Basel). 2020 May 2;11(5):501. doi: 10.3390/genes11050501 (PMC7290959; doi:10.3390/genes11050501)
Supplement: Supplementary file 1 [file genes-11-00501-s001.pdf]

Supplementary Materials

Genetics of Alzheimer's Disease progression

Contents

Supplementary Materials 1

Supplementary 1: Trial Design 2

Figure S1. Study Schematic. 2

Supplementary 2: GWAS Results 3

Table S2a. Candidate Variant Analysis: Change from Baseline in ADAS-cog at Week 48 4

Table S2b. Candidate Variant Analysis: Change from Baseline in CDR-SB at Week 48 5

Figure S2a. QQ plot for ADAS-cog 6

Figure S2b. QQ plot for CDR-SB 6

Supplementary 3: Power Calculations for Association Studies 7

Figure S3. Power curves for candidate and GWAS analysis, for both endpoints 7

Supplementary 4: Detail on Clinical Covariates 8

Table S4a. Effect of Covariates in GWAS: ADAS-cog 8

Table S4b. Effect of Covariates in GWAS: CDRSB 8

Supplementary 5: Power Calculations for PRS 8

Figure S5a. Power Curves for PRS predicting ADAS-cog change 9

Figure S5b. Power Curves for PRS predicting CDR-SB change 10

Supplementary 6: Power Calculations for GCTA 10

Table S6. Power to detect different heritability values 10

Supplementary 7: Details on Genotyping and QC 11

APOE Genotyping 11

Supplementary 8: Mixed Model Analysis 12

Table S8. Results of individual candidate SNP association with progression as assessed by a mixed model with repeated measures 12

Supplementary 9: Quantiles Plots 14

Figure S9. Quantiles plots 14

Table S9. PRS Quantiles 14

Supplementary 10: Interaction Analysis between PRS and Candidate Variants 15

Interaction Analysis Methods 15

Interaction Analysis Results 15

Discussion 15

Table S10. Interaction effects for ADAS-cog Endpoint between PRS and Candidate Variants 16
Supplementary References 17

Supplementary 1: Trial Design

AVA102670 and AVA102672 were international, phase III, multicentre, randomised, double-blind, placebo-controlled, parallel-group studies designed to assess efficacy and safety of rosiglitazone XR (RSG XR) as adjunctive therapy in mild-to-moderate Alzheimer's Disease patients already being treated with an approved acetylcholinesterase inhibitor (AChEI, AVA102670) or donezepil (AVA102672) and stratified by *APOE* e4 allele status.

Full details of the clinical trial design and treatment arms is available on the GSK study register: https://www.gsk-studyregister.com/en/. A schematic representation of the study design is shown in Figure S1.

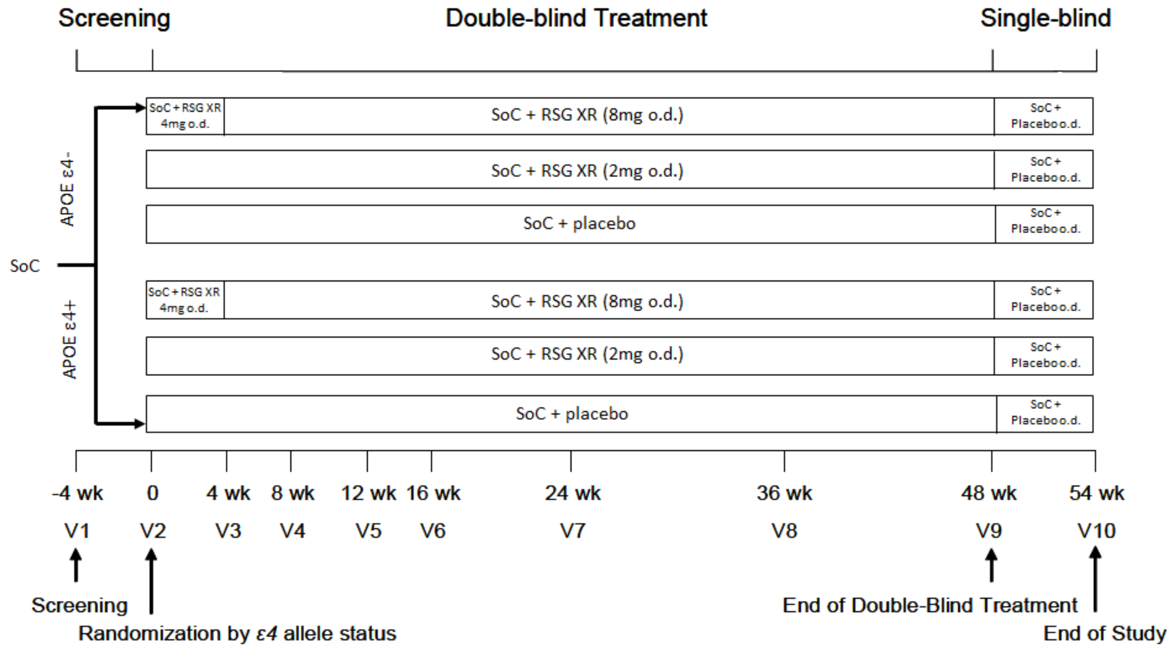

Figure S1. Study Schematic.
Following a 28-day (±7 days) screening period in which patients received standard of care (SoC, AChEI or donezepil), patients were randomised by *APOE* ε4 allele status into one of three arms. Double-blind treatment ended at week 48 with all patients receiving SoC and placebo until study end at week 54.

Supplementary 2: GWAS Results

There were no significant associations with any of the 39 candidate variants, or genome-wide variants with either primary endpoints (Table below). Here a positive effect size/change score represented faster cognitive decline. Significance thresholds were $p<6.41 \times 10^{-4}$ for candidate variants and $p<2.5 \times 10^{-8}$ for genome-wide variants.

Genome-wide differences in allele frequency that correlate with phenotype can be an indicator of various artefacts and are routinely corrected for using genomic control (GC, Dadd *et al* 2009). This process calculates an inflation factor, lambda, which summarises evidence for genome-wide deviation from expected test statistics. A lambda value of 1 indicates no evidence of inflation, and typically values below 1.045 are of no concern. Inflation can be tested by adjusting test statistics and recalculating lambda. Lambda for ADAS-cog before GC adjustment is 0.987308 and 0.987308 after. Lambda for CDR-SB before GC adjustment is 1.000035 and 1 after. This indicates that there is little concern over population structure not captured by Principal Components in this analysis.

The effect estimate is with respect to the minor allele. 24 candidate variants have a MAF>20% (see supplementary Table S2a and S2b)

Candidate variants are sorted by p-value

Table S2a. Candidate Variant Analysis: Change from Baseline in ADAS-cog at Week 48

Variant	Gene	Effect (SE)	p-Value	Effect Allele Frequency
rs2632516	BZRAP1-AS1	-0.07 (0.032)	0.03	0.566
rs77493189	ENSG00000261879;SCIMP	0.096 (0.045)	0.034	0.138
rs3865444	CD33	0.066 (0.034)	0.05	0.301
rs113260531	SCIMP	0.089 (0.046)	0.051	0.133
rs75932628	TREM2	-0.32 (0.191)	0.094	0.007
rs12053868	IL1RAP	0.098 (0.06)	0.102	0.100
rs3795065	CNN2-ABCA7	0.055 (0.034)	0.103	0.353
rs28394864	ABI3	0.05 (0.032)	0.121	0.479
rs59690116	ENSG00000273492-ENSG00000229025	0.049 (0.034)	0.154	0.347
rs9381563	CD2AP	-0.046 (0.033)	0.16	0.367
rs6014724	CASS4	0.075 (0.056)	0.184	0.101
rs7810606	EPHA1	0.04 (0.032)	0.215	0.510
rs6448453	CLNK	-0.041 (0.034)	0.222	0.293
rs11257238	ECHDC3	0.036 (0.032)	0.263	0.385
rs35371668	HLA-DRB1- HLA-DQA1	-0.057 (0.054)	0.287	0.126
rs6931277	HLA-DRB1	-0.05 (0.047)	0.289	0.131
rs117618017	APH1B	-0.058 (0.055)	0.293	0.143
rs114360492	CNTNAP2	-0.66 (0.704)	0.349	0.001
rs184384746	HESX1	-0.5 (0.543)	0.357	0.002
rs10792832	RNU6-560P-ENSG00000254699	-0.029 (0.032)	0.374	0.333
rs4575098	ADAMTS4	0.03 (0.036)	0.408	0.256
rs4308	ACE	0.026 (0.034)	0.444	0.358
rs7657553	HS3ST1	-0.025 (0.035)	0.471	0.288
rs11218343	SORL1	0.059 (0.083)	0.474	0.040
rs4236673	CLU/PTK2B	0.021 (0.032)	0.516	0.366
rs12590654	SLC24A4	-0.019 (0.034)	0.588	0.327
rs442495	ADAM10	-0.016 (0.034)	0.642	0.681
rs76726049	ALPK2	0.082 (0.183)	0.653	0.011
rs2093760	CR1	-0.015 (0.038)	0.7	0.225
rs59735493	KAT8	0.011 (0.035)	0.759	0.289
rs2081545	MS4A6A	-0.008 (0.033)	0.796	0.376
rs10933431	INPPD5	0.009 (0.036)	0.797	0.235
rs111278892	ABCA7	0.009 (0.043)	0.828	0.166
rs4663105	BIN1	0.006 (0.032)	0.855	0.450
rs1582763	MS4A4E-MS4A4A	0.006 (0.032)	0.865	0.363
rs8093731	SUZ12P1	0.031 (0.189)	0.871	0.013
rs1859788	ZCWPW1	-0.006 (0.036)	0.876	0.295
rs41289512	APOE[†]	0.007 (0.061)	0.907	0.071
rs867611	PICALM	0 (0.033)	0.991	0.296

Table S2b. Candidate Variant Analysis: Change from Baseline in CDR-SB at Week 48

Variant	Gene	Effect (SE)	p-Value	Effect Allele Frequency
rs3795065	CNN2-ABCA7	0.08 (0.034)	0.019	0.353
rs111278892	ABCA7	0.099 (0.043)	0.023	0.166
rs77493189	ENSG00000261879;SCIMP	0.098 (0.046)	0.033	0.138
rs113260531	SCIMP	0.089 (0.046)	0.055	0.133
rs2632516	BZRAP1-AS1	-0.061 (0.033)	0.065	0.566
rs11257238	ECHDC3	0.059 (0.033)	0.075	0.385
rs442495	ADAM10	-0.055 (0.034)	0.104	0.681
rs75932628	TREM2	0.298 (0.19)	0.117	0.007
rs1582763	MS4A4E-MS4A4A	-0.045 (0.033)	0.171	0.363
rs59690116	ENSG00000273492-ENSG00000229025	0.046 (0.034)	0.177	0.347
rs4236673	CLU/PTK2B	-0.039 (0.033)	0.229	0.366
rs4663105	BIN1	0.038 (0.032)	0.238	0.450
rs2081545	MS4A6A	-0.036 (0.033)	0.276	0.376
rs4308	ACE	0.036 (0.034)	0.298	0.358
rs10933431	INPPD5	0.034 (0.037)	0.354	0.235
rs1859788	ZCWPW1	-0.031 (0.036)	0.386	0.295
rs28394864	ABI3	-0.027 (0.033)	0.41	0.479
rs7657553	HS3ST1	-0.028 (0.036)	0.434	0.288
rs76726049	ALPK2	-0.136 (0.185)	0.463	0.011
rs117618017	APH1B	-0.041 (0.056)	0.464	0.143
rs12053868	IL1RAP	0.044 (0.061)	0.473	0.100
rs6014724	CASS4	0.04 (0.057)	0.478	0.101
rs114360492	CNTNAP2	-0.456 (0.701)	0.516	0.001
rs41289512	APOE†	-0.04 (0.062)	0.521	0.071
rs12590654	SLC24A4	0.021 (0.035)	0.539	0.327
rs8093731	SUZ12P1	0.114 (0.19)	0.548	0.013
rs867611	PICALM	0.018 (0.034)	0.594	0.296
rs6931277	HLA-DRB1	-0.025 (0.048)	0.606	0.131
rs35371668	HLA-DRB1- HLA-DQA1	-0.028 (0.055)	0.608	0.126
rs7810606	EPHA1	0.014 (0.032)	0.656	0.510
rs10792832	RNU6-560P-ENSG00000254699	0.014 (0.033)	0.666	0.333
rs9381563	CD2AP	0.012 (0.033)	0.718	0.367
rs59735493	KAT8	-0.012 (0.035)	0.729	0.289
rs6448453	CLNK	0.01 (0.034)	0.764	0.293
rs3865444	CD33	-0.009 (0.034)	0.784	0.301
rs184384746	HESX1	-0.072 (0.544)	0.895	0.002
rs4575098	ADAMTS4	-0.003 (0.037)	0.935	0.256
rs11218343	SORL1	-0.006 (0.084)	0.946	0.040
rs2093760	CR1	0 (0.039)	0.999	0.225

†: NB: Phenotype is residualised for *APOE* ε4 allele count; this coefficient is not immediately interpretable

QQ Plots are shown below for each of the GWAS analyses. There was no evidence for test statistic inflation following genomic control, and there are unlikely to be additional genome-wide differences that have not been correctly accounted for by quality control.

Figure S2a. QQ plot for ADAS-cog

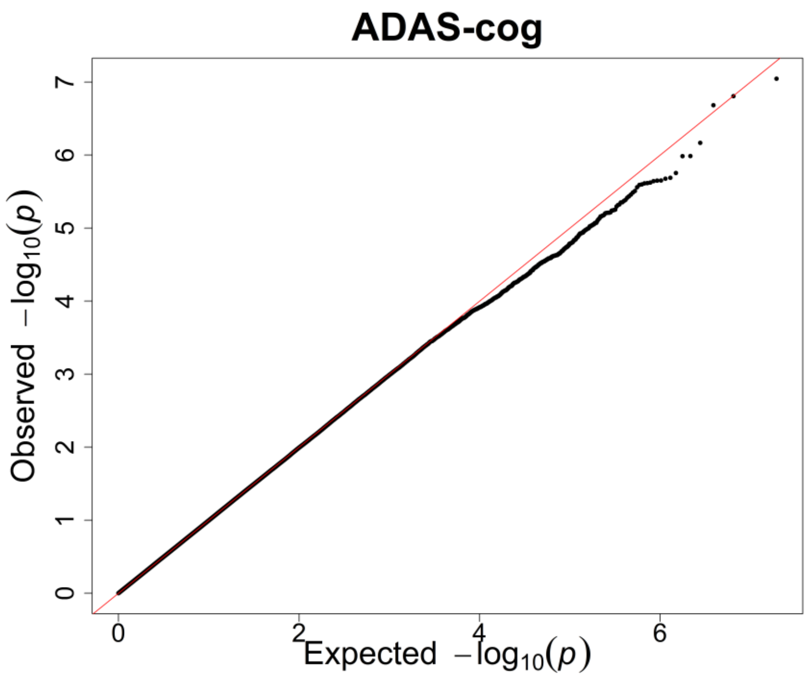

Figure S2b. QQ plot for CDR-SB

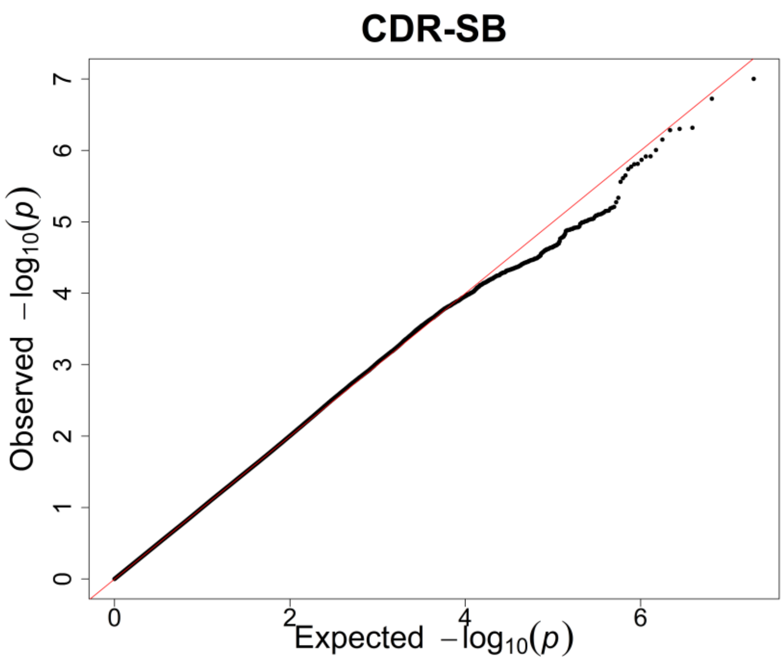

Supplementary 3: Power Calculations for Association Studies

Power calculations for ADAS-Cog change and CDR-SB change from baseline were conducted for the 39 candidate variants and GWAS.

The following parameters were used:

After accounting for missing data,
ADAS-Cog change sample size : 2060
CDR-SB change sample size : 1996

Candidate Variant Minor Allele Frequency Range : 0 to 0.5

ADAS-Cog absolute effect size range: 0 to 6.78 (1 phenotypic SD)
CDR-SB absolute effect size range: 0 to 2.45 (1 phenotypic SD)

Candidate Variant α: $p<6.41 \times 10^{-4}$
GWAS Alpha α: $p<2.5 \times 10^{-8}$

Under a genetic additive model, candidate variants have at least 80% power for ADAS-Cog and CDR-SB change if the absolute effect size is greater than 1.36 and 0.49 phenotypic units respectively and minor allele frequency is >20%. Twenty-four of the 39 candidate variants had minor allele frequencies >20% (Table S2a).

GWAS variants have at least 80% power for ADAS-Cog and CDR-SB change if the absolute effect size is greater than 3.39 and 1.23 phenotypic units respectively and minor allele frequency is >20%.

Figure S3. Power curves for candidate and GWAS analysis, for both endpoints

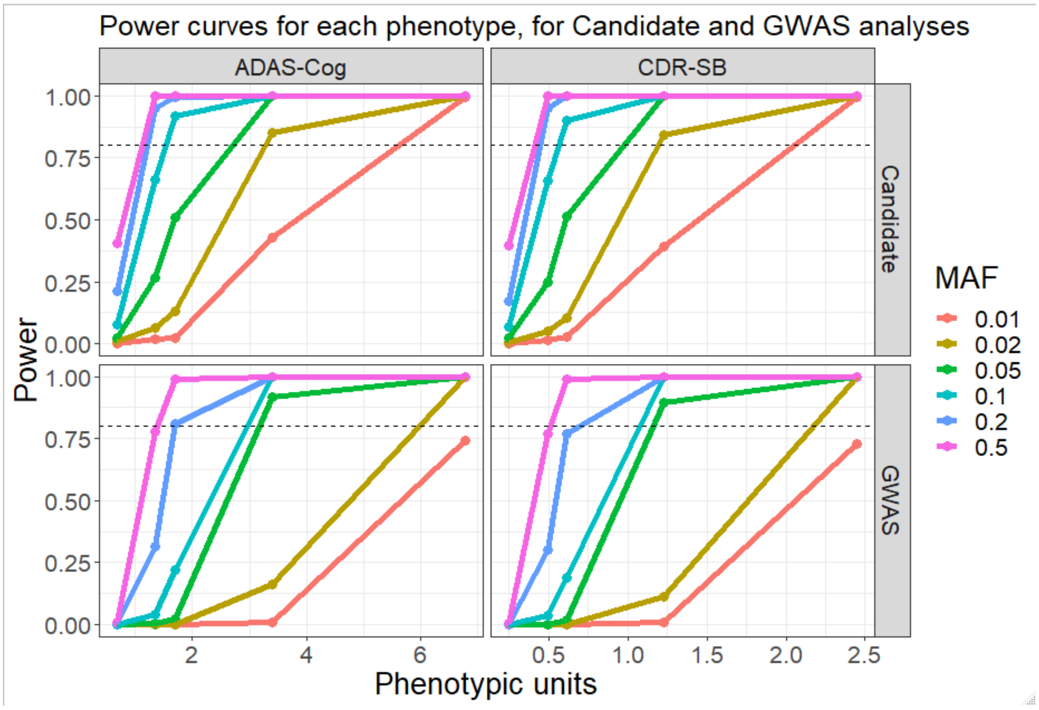

Supplementary 4: Detail on Clinical Covariates

Table S4a. Effect of Covariates in GWAS: ADAS-cog

TERM	ESTIMATE	STD. ERROR	T VALUE	PR(>|T|)
INTERCEPT	10.738	2.008	5.348	p<0.0001
ADAS-COG BASELINE	-0.012	0.023	-0.537	0.591
STUDYID:AVA102672	-1.113	0.300	-3.706	p<0.0001
BMI	-0.078	0.036	-2.177	0.030
TRTGRP:8MGRSGXR	0.331	0.348	0.953	0.341
TRTGRP:PLACEBO	0.399	0.344	1.160	0.246
MMSE TOTAL	-0.517	0.055	-9.350	p<0.0001
YEARS EDUCATION	0.215	0.038	5.676	p<0.0001
TREATMENT DURATION	0.008	0.002	3.925	p<0.0001
APOE ε4 COPIES	0.493	0.210	2.346	0.019
PC1	8.822	6.921	1.275	0.203
PC2	-18.035	7.203	-2.504	0.012
PC3	5.376	6.801	0.791	0.429
PC4	4.864	8.658	0.562	0.574
PC5	-7.330	7.174	-1.022	0.307
PC6	3.076	7.053	0.436	0.663

Table S4b. Effect of Covariates in GWAS: CDRSB

TERM	ESTIMATE	STD. ERROR	T VALUE	PR(>|T|)
INTERCEPT	5.577	0.617	9.044	p<0.0001
CDR-SB BASELINE	-0.121	0.019	-6.427	p<0.0001
STUDYID:AVA102672	-0.434	0.111	-3.919	p<0.0001
BMI	-0.019	0.013	-1.472	0.141
TRTGRP:8MGRSGXR	0.405	0.128	3.162	0.002
TRTGRP:PLACEBO	0.339	0.126	2.680	0.007
MMSE TOTAL	-0.203	0.016	-12.563	p<0.0001
YEARS EDUCATION	0.037	0.014	2.640	0.008
TREATMENT DURATION	0.002	0.001	2.611	0.009
APOE ε4 COPIES	0.166	0.078	2.140	0.033
PC1	3.543	2.549	1.390	0.165
PC2	-6.798	2.640	-2.575	0.010
PC3	5.307	2.522	2.104	0.035
PC4	6.956	3.146	2.211	0.027
PC5	-3.927	2.633	-1.491	0.136
PC6	-3.790	2.593	-1.462	0.144

Supplementary 5: Power Calculations for PRS

Power for PRS was estimated using a series of equations provided by Dudbridge (2013) and implemented in AVENGEME (Palla & Dudbridge 2015). These required several assumptions, so power calculations are presented below on a range of possible values.

Power curves are presented separately for ADAS-Cog and CDR-SB, with P_T and target sample size fixed at those observed in the analysis above. In PRS nomenclature, the GWAS used to select SNPs for prediction was termed ‘base’; the base sample size was 54,162 (31.4% cases) as reported in Lambert *et al* (2013). A prevalence of Alzheimer’s Disease (AD) of 0.712% was assumed (GBD 2016). Power was calculated for an alpha threshold of 0.001 – as was appropriate for PRS based on a simulation study carried out by Euesden *et al* (2014) to account for multiple testing inherent in evaluating PRS at multiple thresholds.

Power was calculated with different values for three parameters. Along the x axis are different values for genetic correlation between base – i.e. GWAS, here Lambert et al 2013 GWAS of AD - and target phenotype – here cognitive decline measured either by ADAS-cog change or CDR-SB change. In colour are different values for the proportion of variance in AD explained by genetic factors – a range of values between 5% and 20% are presented. In each panel are separate calculations, based on different assumptions on the proportion of ***non-causal*** SNPs genome wide for AD. Three estimates are tested – 10% (i.e. most of the genome is causal), 50% (i.e. half of the genome is causal) and 90% (i.e. little of the genome is causal). Power will be higher if the proportion of non-causal SNPs (pi0) is lower – i.e. a larger proportion of the genome is causal.

On the figures below a horizontal dotted line indicates 80% power. In all cases, the calculations indicated that there was good power (>80%) to observe an association between the AD PRS and disease progression (ADAS-Cog and CDR-SB) provided the genetic correlation between the two phenotypes was greater than ~5%.

Figure S5a. Power Curves for PRS predicting ADAS-cog change

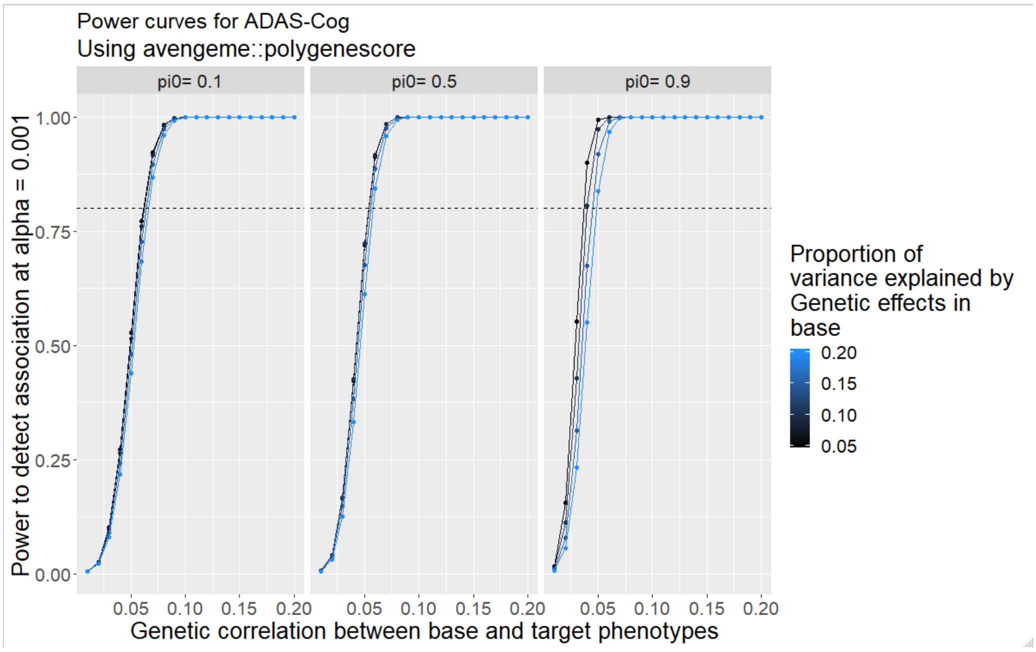

Figure S5b. Power Curves for PRS predicting CDR-SB change

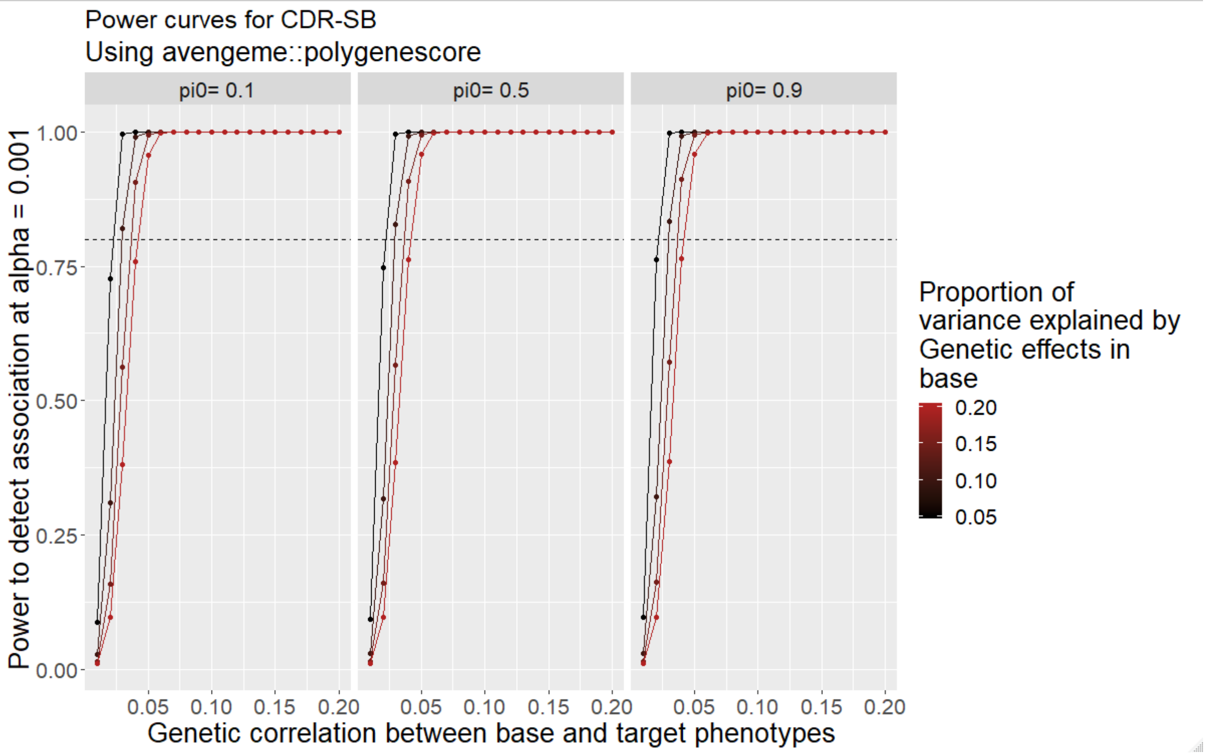

Supplementary 6: Power Calculations for GCTA

Using an online implementation of the formulae in Visscher *et al* (2014) (http://cnsgenomics.com/shiny/gctaPower/), power for GCTA was derived. This calculation required an assumption on the variance of SNP-derived genetic relationships – i.e. the variance in relatedness as measured by DNA, between pairs of individuals. As the current samples were unrelated individuals measured genome-wide, the default value of $2x10^{-5}$ was used. Heritability (h^2) is the proportion of variance in phenotype in a population that can be explained by common SNPs genome-wide. Here, power to detect a range of heritability values was estimated. These results indicated that power to detect even large heritability estimates (20%) was poor given the sample sizes used here.

Table S6. Power to detect different heritability values

	Power for ADAS-cog change (n=2060)	Power for CDR-SB change (n=1996)
h^2 = 5%	6.2%	6.1%
h^2 = 10%	10%	9.7%
h^2 = 20%	25.6%	24.3%

Supplementary 7: Details on Genotyping and QC

Venous blood was collected from each subject who provided written informed consent for genetic research. Genomic DNA was extracted from peripheral blood using the Gentra Puregene kit on the Autopure LS (Qiagen, Valencia, CA) by Quest Diagnostics (Valencia, CA, USA or Heston, UK). Genotyping was performed using the Affymetrix Axiom PMRA (Santa Clara, CA) by the Bioprocessing Solutions Alliance (BSA) under Brooks Automation Inc. (Piscataway, NJ). The array data with a reference set of haplotypes was used to impute variants across the genome including the HLA region.

The genome-wide array data were used to impute gene dosages: For each array, genotypes were aligned to the reference strand and phased by chromosome using sequence and genotype data to estimate haplotypes and unobserved genotypes with HAPI-UR v1.01 (Williams *et al* 2012). The phased haplotypes were used to impute genotype dosages using the 1000 Genomes Project reference haplotypes (phase1_release_v3.20101123 without singletons and the minimac 2012-11-16 release. HIBAG v1.2.4 was used for imputing HLA genotypes (Zheng *et al* 2014). Standard quality control exclusions were applied.

Primary endpoint analyses were conducted on candidate and genome-wide variants with a minor allele frequency ≥0.01 and an imputation r^2 ≥0.30. To supplement the limited number of candidate variants associated with cognitive decline in patients diagnosed with AD, candidate variants from AD susceptibility loci were examined to ascertain their role, if any, in cognitive decline.

APOE Genotyping

APOE genotyping, performed on all subjects to support randomization into the original clinical trials, was performed by Laboratory Corporation of America (Research Triangle Park, North Carolina, USA) based on TaqMan determination of rs7412 and rs429358.

Supplementary 8: Mixed Model Analysis

Congruency between the present data and two recent publications was evaluated using mixed effect models. These publications suggest associations between genetic variants in *IL1RAP* (Ramanan *et al* 2018) and *TREM2* (Del Aguila *et al* 2018) and AD progression. The *IL1RAP* variant was associated with higher rates of amyloid accumulation (independent from *APOE*) and rs12053868-G carriers were more likely to progress from mild cognitive impairment to AD and exhibited greater longitudinal temporal cortex atrophy on magnetic resonance imaging. For *TREM2* Del Aguila report, rs143332484 reached "nominal" significance with progression as measured by CDR-SB (p=0.02) and the Free and Cued Selective Reminding Test (FCSRT)-Free Recall measure of episodic memory (p=0.02). However, the *TREM2* analysis did not include correction for multiple testing. Another *TREM2* variant (rs75932628), not in LD with rs143332484, was previously associated with AD risk (Guerreiro *et al*, 2013), not AD progression. While these publications have limitations to understanding genetic predictors of cognitive decline, we undertook a similar analysis approach using the data here – namely, a linear mixed-model repeated measure (MMRM) framework that incorporates additional cognitive assessment data than simply the change from Baseline to Week 48 approach employed in our main analysis.

There were no significant results with any of the 3 candidate variants. A liberal significance threshold was set at p<0.017 for 3 candidate variants after adjusting for Type I error, to evaluate whether the results of the previous study replicated here.

Table S8. Results of individual candidate SNP association with progression as assessed by a mixed model with repeated measures

Candidate Variant Analysis: ADAS-cog at weeks 0-48 (original analysis)

Variant	Gene	Effect Size	p-Value	SE
rs75932628	*TREM2*	1.605	0.175	1.184
rs143332484	*TREM2*	-0.935	0.479	1.323
rs12053868	*IL1RAP*	0.572	0.126	0.373

Candidate Variant (interaction with visit week) Analysis: ADAS-cog at weeks 0-48 (MMRM)

Variant	Gene	Effect Size	p-Value	SE
rs75932628	*TREM2*	-0.029	0.327	0.029
rs143332484	*TREM2*	-0.070	0.023	0.031
rs12053868	*IL1RAP*	0.019	0.035	0.008

Candidate Variant Analysis: CDR-SB at weeks 0-48 (original analysis)

Variant	Gene	Effect Size	p-Value	SE
rs75932628	*TREM2*	0.672	0.206	0.531
rs143332484	*TREM2*	0.704	0.238	0.597
rs12053868	*IL1RAP*	-0.064	0.705	0.169

Candidate Variant (interaction with visit week) Analysis: CDR-SB at weeks 0-48 (MMRM)

Variant	Gene	Effect Size	p-Value	SE
rs75932628	*TREM2*	0.016	0.137	0.011
rs143332484	*TREM2*	-0.004	0.720	0.012
rs12053868	*IL1RAP*	0.004	0.200	0.003

Supplementary 9: Quantiles Plots

The impact of PRS on phenotype can be interpreted through splitting individuals into quantiles based on their PRS and comparing the average phenotype amongst individuals in each quantile with individuals in a reference quantile. Here, individuals were split into 5 quintiles, based on the AD PRS at the most predictive threshold for each phenotype, and compared with reference to the 3rd quantile, i.e. the middle quantile. This is equivalent to asking how an individual in the top 20% for genetic risk compares to an 'average' individual, and so on. These results are presented below graphically and as tables.

Figure S9. Quantiles plots

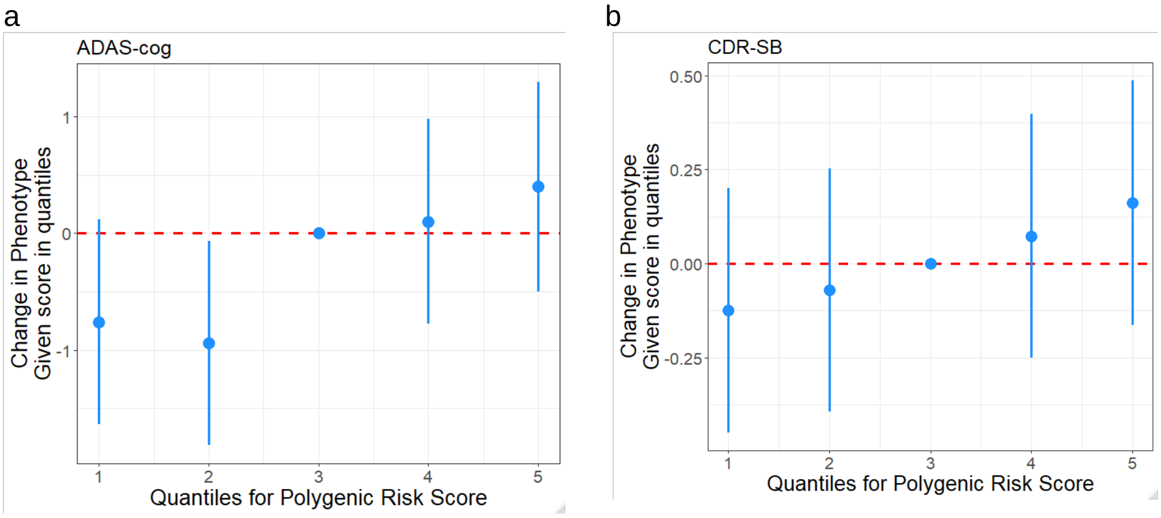

Individuals in each quantile for PRS were compared to individuals in the middle quantile, for ADAS-cog (a) and CDR-SB (b).

Table S9. PRS Quantiles

Mean phenotype change amongst individuals in each quantile for PRS, compared to individuals in the middle quantile as reference

QUANTILE	ADAS-COG COEFFICIENT (95%CI)	CDR-SB COEFFICIENT (95% CI)
**1**	-0.76 (0.12 to -1.64)	-0.12 (0.20 to -0.45)
**2**	-0.94 (-0.06 to -1.82)	-0.07 (0.25 to -0.39)
**3**	Reference	Reference
**4**	0.10 (0.98 to -0.77)	0.07 (0.40 to -0.25)
**5**	0.40 (1.30 to -0.50)	0.16 (0.49 to -0.16)

In both cases, individuals in the lowest PRS quantile (quantile 1 - i.e. the bottom 20% for PRS, had nominally lower ADAS-cog changes than individuals in the middle quantile. High PRS individuals in the highest PRS quantile (quantile 5 – the top 20% for PRS) had higher ADAS-cog changes than individuals in the middle quantile. CDR-SB results trended in the same direction across quantiles – CDR-SB change is nominally lower in the bottom PRS quantile - and highest in the top PRS quantile - vs the middle PRS quantile.

Supplementary 10: Interaction Analysis between PRS and Candidate Variants

Interaction Analysis Methods

The interaction between genotype at a single SNP and PRS was investigated, for each of 39 candidate variants. This analysis sought to test whether candidate variants have a stronger effect in otherwise genetically low-risk individuals, and explore genetic heterogeneity in AD.

Any PRS may contain variants in linkage disequilibrium (LD) with the candidate SNP being tested and this must be avoided. For each candidate SNP, the region 250kb either side of that variant was excluded prior to PRS calculation, leading to a separate PRS for each candidate SNP*PRS interaction tested. PRS was calculated at the most predictive threshold from the genome-wide PRS model above.

Interaction Analysis Results

Interaction analysis was performed for ADAS-cog only, as there was not a significant main effect of PRS predicting CDR-SB change. The interaction analysis for ADAS-cog showed no significant interaction effects – the two variants with the lowest *p*-values were rs114360492 in the locus of *CNTNAP2* (*Contactin Associated Protein Like 2)* (*p*=0.024) and rs1582763 in the locus of *MS4A4E-4A* (Membrane Spanning 4-Domains A4E-4A) (*p*=0.027), which did not meet statistical significance. While the *APOE* ε*4* allele is an important contributor to AD risk, there was no evidence for an interaction effect between PRS and *APOE* genotype at rs41289512 (*p*=0.88) in ADAS-cog score change.

Discussion

The table below shows the results for the interaction term for PRS by candidate genotype regressed on ADAS-Cog endpoint in a linear model with clinical covariates used earlier and 6 PCs.

This analysis applied novel approaches to assess the heritability of cognitive decline – evaluating PRS by candidate SNP interaction and genome-wide data to assess the heritability of cognitive decline.

The interaction analysis for ADAS-Cog showed no significant interaction effects – the two variants with the lowest p-values were *CNTNAP2* (P=0.024) and MS4A4E-MS4A4A (P=0.027), which did not meet our statistical significance threshold. While *APOE* is an important contributor to AD risk, there was no evidence for an interaction effect between PRS and *APOE* (p=0.88) in ADAS-cog change score.

Table S10. Interaction effects for ADAS-cog Endpoint between PRS and Candidate Variants

Gene	Chr	Hg19 position	Coefficient Beta (larger positive values indicate 'worsening')	SE	p-value	Low-CI	Hi-CI
ADAMTS4	1	161155392	0.011	0.233	0.962	-0.445	0.468
CR1	1	207786828	-0.021	0.25	0.932	-0.511	0.468
BIN1	2	127891427	0.167	0.204	0.411	-0.232	0.566
INPPD5	2	233981912	-0.098	0.238	0.679	-0.565	0.368
HESX1	3	57226150	4.403	3.449	0.202	-2.356	11.163
IL1RAP	3	190300004	0.466	0.401	0.245	-0.32	1.252
CLNK	4	11026028	0.032	0.222	0.887	-0.404	0.468
HS3ST1	4	11723235	-0.141	0.221	0.522	-0.574	0.291
HLA-DRB1	6	32583357	-0.025	0.33	0.939	-0.673	0.622
HLA-DRB1-HLA-DQA1	6	32561638	-0.079	0.377	0.834	-0.817	0.659
TREM2	6	41129252	-1.716	1.411	0.224	-4.482	1.051
CD2AP	6	47432637	0.103	0.22	0.641	-0.328	0.534
ZCWPW1	7	99971834	-0.083	0.226	0.714	-0.526	0.36
CNTNAP2	7	145950029	14.925	6.603	0.024	1.983	27.867
EPHA1	7	143108158	-0.115	0.202	0.568	-0.51	0.28
CLU/PTK2B	8	27464929	-0.269	0.216	0.213	-0.691	0.154
ECHDC3	10	11717397	0.306	0.209	0.145	-0.105	0.716
MS4A6A	11	59958380	-0.293	0.219	0.18	-0.722	0.135
MS4A4E-MS4A4A	11	60021948	-0.482	0.218	0.027	-0.91	-0.055
PICALM	11	85776544	-0.121	0.213	0.57	-0.538	0.296
RNU6-560P-ENSG00000254699	11	85867875	-0.192	0.202	0.343	-0.589	0.205
SORL1	11	121435587	0.28	0.584	0.632	-0.865	1.424
SLC24A4	14	92938855	-0.087	0.217	0.687	-0.512	0.337
ADAM10	15	59022615	0.277	0.219	0.206	-0.152	0.706
APH1B	15	63569902	-0.161	0.361	0.657	-0.868	0.547
KAT8	16	31133100	-0.277	0.225	0.219	-0.718	0.164
SCIMP	17	5138980	0.38	0.3	0.206	-0.209	0.969
[ENSG00000261879;SCIMP	17	5118951	0.309	0.292	0.29	-0.263	0.881
ABI3	17	47450775	-0.143	0.211	0.499	-0.556	0.271
[ACE]	17	61559625	-0.295	0.225	0.189	-0.736	0.145
BZRAP1-AS1	17	56409089	-0.13	0.208	0.53	-0.537	0.276
SUZ12P1	18	29088958	-0.305	1.419	0.83	-3.086	2.475
ALPK2	18	56189459	0.582	1.244	0.64	-1.857	3.022
ABCA7	19	1039323	-0.364	0.289	0.208	-0.93	0.202
CNN2-ABCA7	19	1039444	0.057	0.214	0.791	-0.362	0.475
APOE	19	45351516	-0.06	0.4	0.88	-0.845	0.724
CD33	19	51727962	-0.33	0.213	0.121	-0.748	0.087
CASS4	20	54998544	0.431	0.335	0.199	-0.226	1.087

Supplementary References

Dadd, T., Weale, M. E., & Lewis, C. M. (2009). A critical evaluation of genomic control methods for genetic association studies. *Genetic Epidemiology: The Official Publication of the International Genetic Epidemiology Society*, *33*(4), 290-298.

Del-Aguila JL, Fernandez MV, Schindler S et al. (2018) Assessment of the genetic architecture of Alzheimer's disease risk in rate of memory decline. *J Alzheimer's Disease* **62**: 745-56.

Dudbridge F. (2013). Power and predictive accuracy of polygenic risk scores. *PLoS genetics*, *9*(3), e1003348. doi:10.1371/journal.pgen.1003348

Euesden, J., Lewis, C. M., & O'Reilly, P. F. (2014). PRSice: polygenic risk score software. *Bioinformatics*, ***31*(9),** 1466-1468.

GBD 2016 Neurology Collaborators (2019). Global, regional, and national burden of neurological disorders, 1990-2016: a systematic analysis for the Global Burden of Disease Study 2016. *The Lancet. Neurology*, ***18*(5),** 459–480.

Guerreiro R, Wojtas A, Bras J et al. (2013) TREM2 variants in Alzheimer's disease. *N Engl J Med* **368**: 117-27.

Howie B, Fuchsberger C, Stephens M, Marchini J, Abecasis GR. Fast and accurate genotype imputation in genome -wide association studies through pre-phasing. Nature Genetics 2012; **44**: 955-959

Li Y, Willer CJ, Sanna S, Abecasis GR. Genotype imputation. Annual Review Genomics and Human Genetics 2009; **10**: 387-406.Palla, L., & Dudbridge, F. (2015). A Fast Method that Uses Polygenic Scores to Estimate the Variance Explained by Genome-wide Marker Panels and the Proportion of Variants Affecting a Trait. *American journal of human genetics*, *97*(2), 250–259. doi:10.1016/j.ajhg.2015.06.005

Ramanan VK, Risacher SL, Nho K et al (2015) GWAS of longitudinal amyloid accumulation on ^{18}F-florbetapir PET in Alzheimer's disease implicates microglial activation gene *IL1RAP*. *Brain* **138**: 3076-88.

Visscher et al. (2014) Statistical power to detect genetic (co)variance of complex traits using SNP data in unrelated samples. PLoS Genetics, 10(4): e1004269.

Williams AL, Patterson N, Glessner J, Hakonarson H, Reich D. Phasing of many thousands of genotyped samples. Am J Hum Genet. 2012 Aug 10;91(2):238-51

Zheng X, Shen J, Cox C, Wakefield JC, Ehm MG, Nelson MR, Weir BS. HIBAG--HLA genotype imputation with attribute bagging. Pharmacogenomics J. 2014 Apr;14(2):192-200
